# Independence from Kinetoplast DNA Maintenance and Expression Is Associated with Multidrug Resistance in Trypanosoma brucei *In Vitro*

**DOI:** 10.1128/AAC.00122-14

**Published:** 2014-05

**Authors:** Matthew K. Gould, Achim Schnaufer

**Affiliations:** Centre for Immunity, Infection & Evolution, Institute of Immunology & Infection Research, University of Edinburgh, Edinburgh, United Kingdom

## Abstract

It is well known that several antitrypanosomatid drugs accumulate in the parasite's mitochondrion, where they often bind to the organellar DNA, the kinetoplast. To what extent this property relates to the mode of action of these compounds has remained largely unquantified. Here we show that single point mutations that remove the dependence of laboratory strains of the sleeping sickness parasite Trypanosoma brucei on a functional kinetoplast result in significant resistance to the diamidine and phenanthridine drug classes.

## TEXT

Trypanosomes are protist parasites that are the causative agents of a range of pathogenic infections in humans and in animals. A defining characteristic of these organisms is the presence of the kinetoplast, a unique structure comprising the cell's mitochondrial genome (kDNA). In the disease-causing long slender bloodstream forms (BF) of Trypanosoma brucei, subspecies of which cause human African trypanosomiasis (HAT) (or sleeping sickness, caused by T. brucei rhodesiense and T. brucei gambiense) and animal African trypanosomiasis (AAT) (caused by T. brucei brucei), the presence of a functional kinetoplast is essential (1–3). However, the closely related animal pathogens Trypanosoma equiperdum and Trypanosoma evansi are dyskinetoplastic (DK) (i.e., lacking all or critical parts of their kDNA), with no apparent detrimental effect (4, 5). Single point mutations had been identified in the nuclearly encoded subunit γ of the mitochondrial F_1_F_0_-ATPase of the naturally DK trypanosomes T. evansi and T. equiperdum (6) and in a laboratory strain of T. brucei brucei (7) that was chemically induced to lose its kinetoplast through long-term exposure to the DNA intercalator acriflavine (8). We recently reported that some of these mutations are sufficient to fully compensate for the complete loss of kDNA in BF T. brucei (9). The mechanism involves F_0_-independent generation of the essential mitochondrial membrane potential (Δψm), obviating the need for expression of the kDNA-encoded F_0_ subunit *a* (9).

A number of current and potential trypanocidal chemotherapies, and their related compounds, have been shown to accumulate in the mitochondrion and/or interact with kDNA (10–13). Other compounds have been proposed to act, at least in part, by disrupting the replication of kDNA through the inhibition of key enzymes, such as the topoisomerases (14–18). Until now, attempts to quantify the degree to which compounds target the kinetoplast have relied upon comparing the sensitivities of divergent strains and species (16, 19–21). Consequently, the ability to draw definitive conclusions was limited by unknown effects of interstrain/species variations acquired through differing evolutionary selection pressures or culturing histories.

We generated BF T. brucei brucei strain Lister 427 trypanosomes ectopically expressing F_1_F_0_-ATPase subunit γ with an L262P or A281del mutation with both endogenous alleles knocked out. We generated a reference cell line that constitutively expressed ectopic wild-type (WT) subunit γ in a double-endogenous knockout background. DK versions of each mutant γ-expressing cell line were obtained by exposure to 20 nM acriflavine for 7 days (9).

The generation of otherwise isogenic cell lines, either dependent or not on functional kDNA, allowed us to conclusively establish to what extent the mode of action of antitrypanosomatid compounds is related to the parasite's dependence on kDNA maintenance or expression. The 50% effective concentrations (EC_50_s) were determined for a panel of trypanocidal compounds using a slightly modified version of the alamarBlue method (22) as described previously (9).

For the topoisomerase inhibitors etoposide, camptothecin, norfloxacin, and enoxacin, no major change in sensitivity was observed in the kinetoplast-independent γL262P-expressing trypanosomes, either with or without the kinetoplast, compared to that of the γWT-expressing controls (see Table S1 in the supplemental material). These data suggest that for these compounds, the dominant mode of action is not related to kDNA maintenance or expression, which contrasts with a study in Trypanosoma cruzi that reported a kDNA-specific effect for norfloxacin and enoxacin (17). Similarly, for the antimalarial atovaquone, a compound that targets the mitochondrial cytochrome *bc*_1_ complex in Plasmodium falciparum (23), no differences in sensitivity were observed (see Table S1). This is not surprising, since this complex is not expressed in BF T. brucei (24). Interestingly, proguanil, a compound that acts synergistically with atovaquone, presumably by inhibiting an alternative mode of generating the essential mitochondrial membrane potential (Δψm) that is independent of the mitochondrial electron transport chain (23), also displayed no differences in potency. Hence, the alternative mechanism for generating the Δψm that operates in DK trypanosomes is sufficiently different from the one in atovaquone-treated Plasmodium to be insensitive to proguanil.

Of the compounds tested that are currently used to treat HAT and AAT, nifurtimox, suramin, and melarsen oxide showed no kinetoplast-specific activity (see Table S2 in the supplemental material); however, major resistance to the diamidine and phenanthridine trypanocide classes was displayed. For pentamidine, minor resistance factors of 2.7 and 2.5 for γL262P-expressing trypanosomes with and without kDNA, respectively, were observed (Table 1). The resistance to diminazene was more pronounced, resulting in average EC_50_s that were higher by factors of 8.1 or 8.5 (with or without kinetoplast, respectively) (Table 1). Likewise, for the fluorescing DNA stain 4′,6-diamidino-2-phenylindole (DAPI), also a diamidine, resistance factors of 32.0 and 31.5, respectively, were displayed by the same mutant cell lines (Table 1). The highest resistance shown by γL262P-expressing cell lines for any of the diamidine compounds tested was for DB829, with resistance factors of 35.5 and 41.7 (with and without kinetoplast, respectively) (Table 1).

**TABLE 1:** Cross-resistance characterization of L262P or A281del mutated ATP synthase γ expressing trypanosomes with both endogenous alleles knocked out, with or without (DK) their kinetoplast, compared to WT ATP synthase γ-expressing control^*a*^

Compound^*b*^	Lister 427 EC_50_ (mean ± SEM) (nM)	γWT EC_50_ (mean ± SEM) (nM)	γL262P		γL262P (DK)			γA281del		γA281del (DK)		
			EC_50_ (mean ± SEM) (nM)	Resistance factor vs γWT	EC_50_ (mean ± SEM) (nM)	Resistance factor vs γWT	Resistance factor vs γL262P	EC_50_ (mean ± SEM) (nM)	Resistance factor vs γWT	EC_50_ (mean ± SEM) (nM)	Resistance factor vs γWT	Resistance factor vs γA281del
Acriflavine	2.1 ± 0.1	2.9 ± 0.2	17.6 ± 0.5	6.1**	16.1 ± 0.4	5.6***	0.9	3.8 ± 0.6	1.3	44.8 ± 6.6	15.4*	11.8*
Diamidines												
DB75	5.0 ± 0.3	5.0 ± 0.1	15.6 ± 1.2	3.1**	18.2 ± 0.7	3.6**	1.2	3.9 ± 0.5	0.8	2.7 ± 1.1	0.5	0.7
DB829	35 ± 6	23 ± 2	817 ± 196	35.5*	958 ± 167	41.7*	1.2	64 ± 4	2.8***	1047 ± 235	45.5*	16.4*
Pentamidine	0.8 ± 0.2	1.0 ± 0.2	2.7 ± 0.2	2.7*	2.5 ± 0.4	2.5*	0.9	2.0 ± 0.2	2.0*	6.3 ± 0.5	6.3***	3.2**
Diminazene	5.0 ± 0.7	3.4 ± 0.4	27.6 ± 5.7	8.1*	28.8 ± 5.1	8.5*	1.0	8.3 ± 0.7	2.4**	59.7 ± 6.5	17.6**	7.2*
DAPI	6.3 ± 0.1	3.1 ± 0.2	99.3 ± 8.3	32.0**	97.8 ± 8.9	31.5**	1.0	5.8 ± 0.1	1.9	274 ± 3	88.4***	47.2***
Phenanthridines												
Isometamidium	0.32 ± 0.01	0.40 ± 0.02	32.9 ± 3.7	82.3**	63.9 ± 2.1	159.8***	1.9**	0.32 ± 0.04	0.8	198 ± 6	495.0***	618.8***
Ethidium bromide	0.9 ± 0.1	1.1 ± 0.1	239 ± 8	217.3***	318 ± 5	289.1***	1.3**	0.7 ± 0.1	0.6	613 ± 20	557.3***	875.7***
Dihydroethidium	2.6 ± 0.5	0.8 ± 0.1	112 ± 14	140.0*	208 ± 13	260.0**	1.9*	0.9 ± 0.1	1.1***	833 ± 28	1041.3***	925.6***

a Asterisks indicate statistically significant resistance, as calculated using a one-tailed Student's *t* test: *, *P* < 0.05; **, *P* < 0.01; ***, *P* < 0.001.

b The DB compounds and isometamidium were kind gifts from David Boykin and Michael Barrett/Harry de Koning, respectively. All other compounds were purchased from Sigma.

Even greater resistance was displayed by the γL262P mutants to the phenanthridine class of drugs, used for AAT chemotherapy (25). The average EC_50_s for γL262P-expressing cells that still retained a kinetoplast were higher by factors of 82.3, 217.3, and 140.0 for isometamidium, ethidium bromide (EtBr), and dihydroethidium, respectively (Table 1). These EC_50_s (derived from a modified 3-day alamarBlue assay) were confirmed to be broadly representative of the long-term effects on parasite growth by conducting cumulative growth curves over 7 days in the presence of various concentrations of isometamidium for γL262P and γWT-expressing trypanosomes (see Fig. S1A and B in the supplemental material). In the DK version of the γL262P mutants, the resistance factors for the phenanthridines were significantly increased by a further 1.9- (isometamidium), 1.3- (EtBr), and 1.9-fold (dihydroethidium), suggesting that the potency of this compound class is slightly enhanced by the presence of kDNA, even though the cells are no longer dependent on its expression. Exposure to diamidines and phenanthridines invariably resulted in kDNA loss (data not shown), confirming the reported interference of these compounds with kDNA maintenance (12, 14–18). The effect of EtBr on kDNA was investigated in great detail and was shown to inhibit replication initiation of free minicircles (16).

T. brucei cells expressing the γA281del mutation retain some dependence on a functional kinetoplast, at least *in vitro* (9); however, after a period of ∼7 days in the presence of acriflavine, the mutants can adapt to the kinetoplast loss (9). This requirement for additional adaptations in the γA281del cell line is consistent with the generally low level of resistance displayed against the diamidine and phenanthridine compounds, compared to its DK version or the γL262P cell lines (Table 1). Since the alamarBlue assay is conducted over only 3 days, the kinetoplast-retaining γA281del mutants do not have enough time to adapt to test compound-induced loss of kinetoplast expression and consequently have EC_50_s close to those of γWT-expressing trypanosomes.

The lack of resistance to DB75 by the γA281del cell lines (with or without kinetoplast) mirrors the minor resistances presented by the γL262P-expressing trypanosomes, in contrast to the major resistance to DB829 that was observed. DB829 and its prodrug version DB868 are currently under intensive development as potential new chemotherapies for stage II HAT (26–28). Interestingly, the only difference between DB75 and DB829 is the substitution in DB829 of one carbon in each of the two benzene rings for nitrogen (26). This single change partially links the mode of action of DB829 to the parasite's dependence on kDNA.

**FIG 1:**
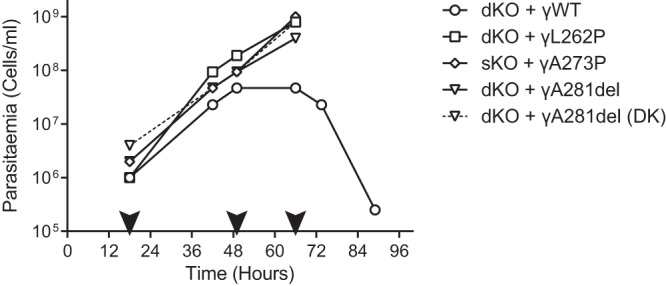
Subunit γ mutations that can compensate for kDNA loss in BF T. brucei brucei also confer drug resistance *in vivo. In vivo* efficacy of EtBr against BF trypanosomes ectopically expressing a WT, L262P, or γA281del allele, with both endogenous alleles knocked out (dKO), or γA273P in a single endogenous knockout background (sKO), was measured by determining parasitemia in blood samples of infected mice. For the A281del-expressing cells, the acriflavine-induced DK form was assayed in parallel (dashed line). The black arrowheads indicate time points of intraperitoneal administration of 10 mg/kg of body weight EtBr to each surviving mouse.

In order to test whether the cell lines with mutated ATP synthase γ remained viable *in vivo* and still retained drug resistance, MF1 mice were inoculated intraperitoneally with each of the cell lines that had been tested *in vitro* (1 × 10^6^ trypanosomes per infection) and treated with normally curative doses of EtBr (Fig. 1; see also Fig. S2 in the supplemental material). For γWT-expressing cells, parasitemia in the mice peaked around 48 h postinoculation, and trypanosomes were entirely absent from the blood after 4 days (by microscopic inspection of blood smears [29]). In contrast, the mutant γ-expressing cell lines were apparently unaffected by EtBr treatment, with parasitemia increasing exponentially until the experiment was ended, clearly demonstrating that trypanosomes with single point mutations in ATP synthase γ that circumvent dependence on kDNA are viable and drug resistant in animal models. *In vivo*, no noticeable adaptation period was apparent upon EtBr-induced loss of the kinetoplast for the γA281del mutant, with parasitemia progressing at rates similar to those for the other mutants and with no discernible difference to that of its DK cell line (Fig. 1; see also Fig. S2). This suggests that, *in vivo*, either the A281del mutation is fully sufficient to compensate for kDNA loss or any secondary adaptation occurs much faster than *in vitro*. A third mutation conferring independence from the kinetoplast, γA273P (in this case, ectopically expressed in a single endogenous knockout background) (9), showed resistance to EtBr similar to that of the γWT-expressing cell line.

Finally, we investigated the possibility of a change in drug accumulation being responsible for the observed resistance phenotypes. Whole-cell uptake of the naturally fluorescent phenanthridines isometamidium and EtBr was monitored using a previously described oil-stop method to halt uptake at specific time points (30). Over 20 min, no significant differences were observed in the rates of uptake of either compound by γL262P-expressing trypanosomes (with or without kinetoplast) compared to that of γWT-expressing trypanosomes (see Fig. S3 in the supplemental material). Thus, mutations to ATP synthase γ do not affect the overall uptake of phenanthridines into the cell, although differences in the rate of accumulation by the mitochondrion cannot be ruled out.

In summary, we have exploited single point mutations in the F_1_F_0_-ATPase to demonstrate that the uncoupling of viability of laboratory strains of T. brucei from a functional kinetoplast results in >80-fold resistance to isometamidium, >200-fold resistance to EtBr, and >30-fold resistance to some diamidines. While an obvious explanation for these findings could be that these drugs interfere with the maintenance and/or expression of kinetoplast-encoding genes, we presently cannot rule out that they have additional, relevant mitochondrial targets and that the altered mechanisms for generating a mitochondrial membrane potential (Δψm) in the mutant cells affect drug accumulation in the mitochondrion. Although the overall drug accumulation of at least phenanthridines was unaffected, a potentially lower Δψm in cells expressing the L262P or A281del mutation could change the intracellular drug distribution. This requires further study.

It seems likely that the levels of resistance we observed can be relevant in the field. For example, it has been reported that Chinese T. evansi isolates (most, if not all, of which have the A281del mutation) have innate resistance to isometamidium (31), and our results strongly suggest that independence from the kinetoplast plays an important role in this resistance. A report concluding that dyskinetoplastidy is not associated with isometamidium resistance was misled by comparing the sensitivities of T. evansi and T. equiperdum strains before and after complete deletion of kDNA (21). As we now know, all strains of these species are already preadapted to kDNA loss (9); whether certain parts of kDNA remain or not is largely irrelevant for phenanthridine resistance.

## Supplementary Material

Supplemental material
