# Supplementary material for: Independence from Kinetoplast DNA Maintenance and Expression Is Associated with Multidrug Resistance in Trypanosoma brucei In Vitro
Source: Antimicrob Agents Chemother. 2014 May;58(5):2925–8. doi: 10.1128/AAC.00122-14 (PMC3993240; doi:10.1128/AAC.00122-14)
Supplement: Supplemental material [file supp_58_5_2925__index.html]

Independence from Kinetoplast DNA Maintenance and Expression Is Associated with Multidrug Resistance in Trypanosoma brucei In Vitro — Supplemental material 

# Independence from Kinetoplast DNA Maintenance and Expression Is Associated with Multidrug Resistance in Trypanosoma brucei*In Vitro*

## Supplemental material

**Files in this Data Supplement:**

- Supplemental file 1 -

  Supplemental Figures S1 to S3 and Tables S1 and S2.

  PDF, 523K
