# Supplementary material for: Independence from Kinetoplast DNA Maintenance and Expression Is Associated with Multidrug Resistance in Trypanosoma brucei In Vitro
Source: Antimicrob Agents Chemother. 2014 May;58(5):2925–8. doi: 10.1128/AAC.00122-14 (PMC3993240; doi:10.1128/AAC.00122-14)
Supplement: Supplemental material [file AAC.00122-14_zac005142794so1.pdf]

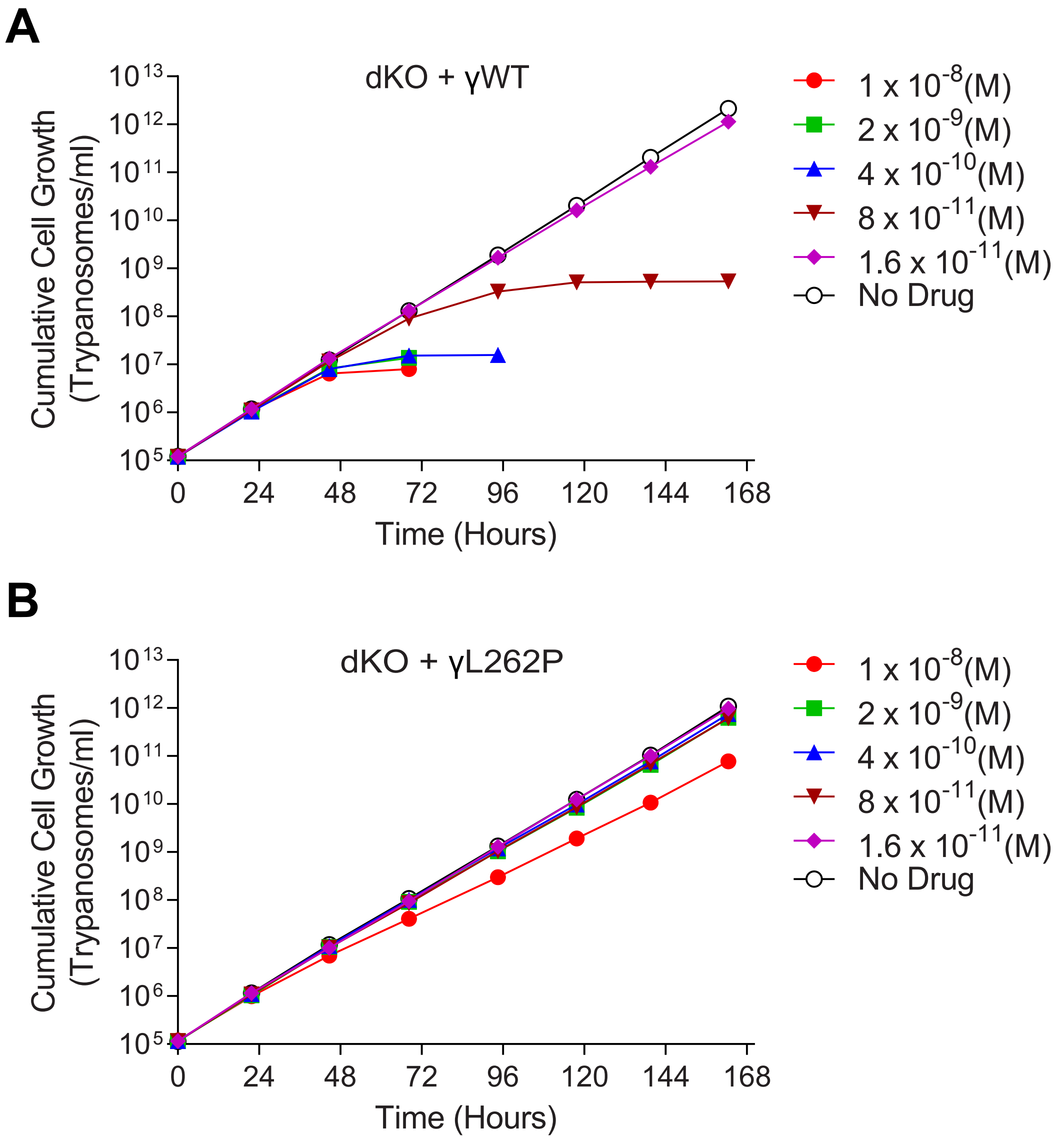

FIG S1 Mutations to ATP synthase γ give resistance to isometamidium *in vitro*. Sensitivity to isometamidium of BF *T. b. brucei* ectopically expressing wild type (A) or L262P mutated (B) ATP synthase γ with both endogenous alleles knocked out was determined by culturing parasites in the concentrations indicated and measuring parasite density every 24 hours. Cumulative cell numbers reflect normalization for dilution during cultivation.

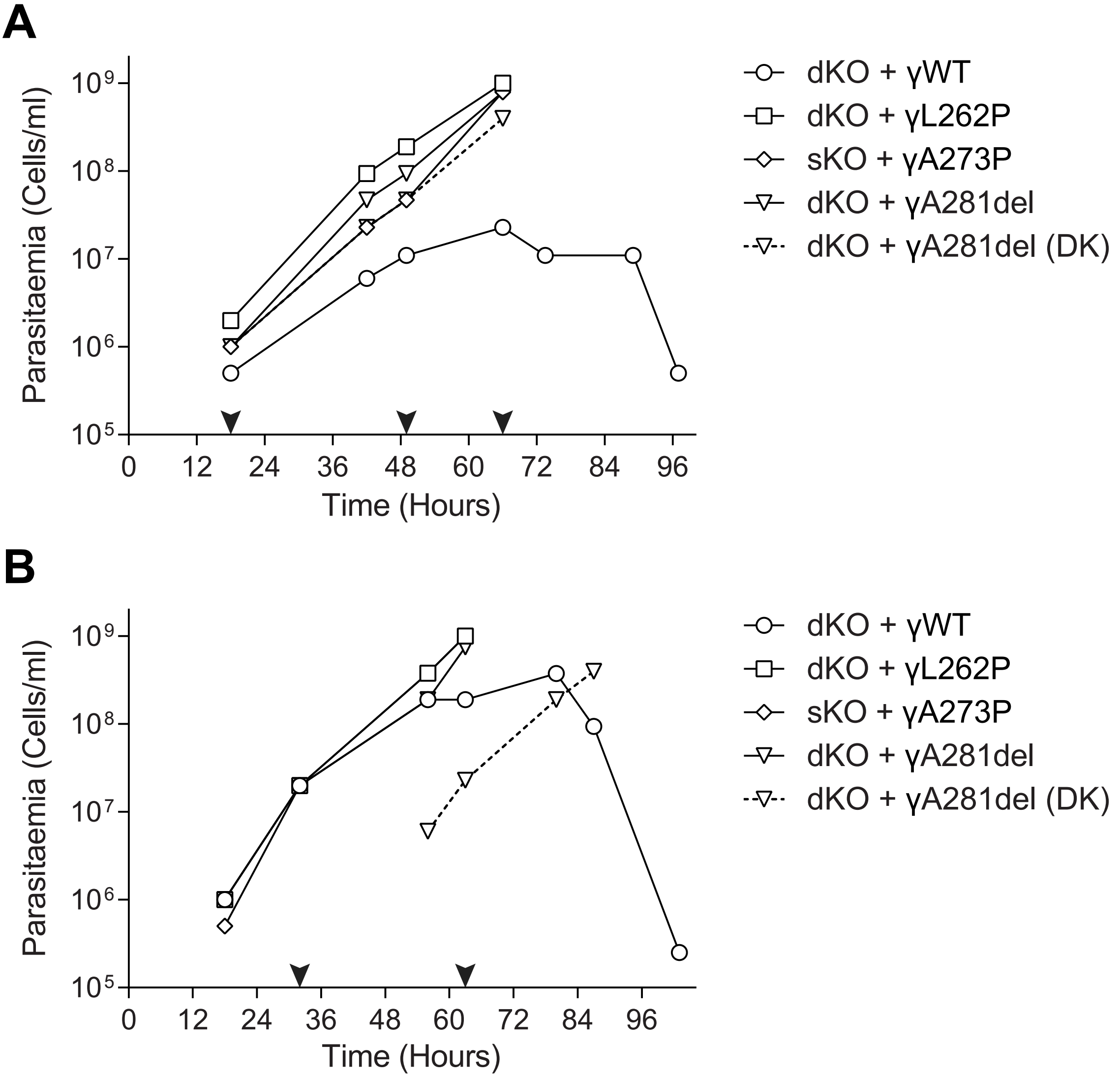

FIG S2 Repeat cohorts of FIG 1: Subunit γ mutations that can compensate for kDNA loss in BF *T. b. brucei* also confer drug resistance *in vivo*. *In vivo* efficacy of EtBr treatment of mice against BF trypanosomes ectopically expressing one of WT, L262P or A281del γ alleles, with both endogenous alleles knocked out, or an A273Pγ in a single endogenous knock out background, was measured by determining parasitaemia in blood samples of infected mice. For the A281del expressing cells, the acriflavine-induced DK form was assayed in parallel (dashed line). The black arrowheads indicate time-points of intraperitoneal administration of 10 mg/kg EtBr to each surviving mouse.

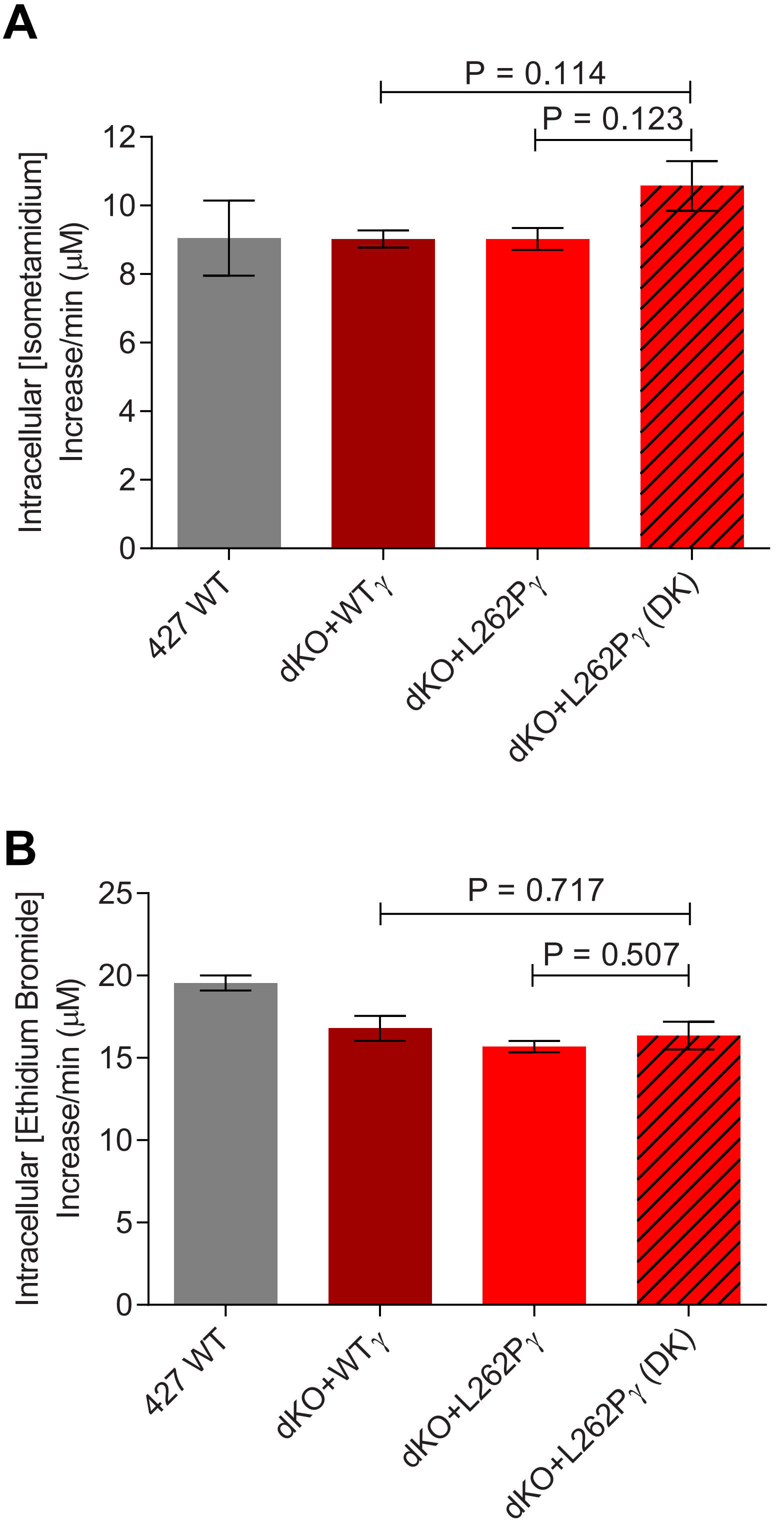

FIG S3 Mutations to ATP synthase γ do not significantly alter the cellular rate of uptake of phenanthridine compounds by BF *T. b. brucei*. Uptake of 10 µM isometamidium (A) and EtBr (B) was measured over 20 minutes in BF trypanosomes ectopically expressing wild type (WT) or L262P mutated ATP synthase γ with both endogenous alleles knocked out. The DK version of the L262P mutant is also included, as is the parental WT Lister 427 cell line. P-values were derived using a paired, 2-tailed Student's t-test.

TABLE S1 Cross resistance characterisation of L262P mutated ATP synthase γ expressing trypanosomes with both endogenous alleles knocked out, with and without (DK) their kinetoplast, compared to WT ATP synthase γ expressing cells.

	Lister 427	γWT	γL262P		γL262P (DK)		
Compound	Mean EC_{50} (μM)	Mean EC_{50} (μM)	Mean EC_{50} (μM)	Resistance Factor *vs.* γWT	Mean EC_{50} (μM)	Resistance Factor *vs.* γWT	Resistance Factor *vs.* γL262P
Topoisomerase inhibitors							
Etoposide	4.74 ± 2.76	1.34 ± 0.08	1.58 ± 0.13	1.2	1.22 ± 0.08	0.9	0.8
Camptothecin	1.13 ± 0.03	0.82 ± 0.01	0.84 ± 0.01	1.0	0.74 ± 0.02	0.9	0.9
Norfloxacin	35.3 ± 1.8	48.9 ± 1.8	45.0 ± 1.9	0.9	44.1 ± 1.6	0.9	1.0
Enoxacin	52.6 ± 1.9	57.1 ± 3.0	52.0 ± 3.2	0.9	50.9 ± 2.3	0.9	1.0
Mitochondria-targeting anti-malarials							
Proguanil	25.1 ± 3.0	22.8 ± 2.1	25.0 ± 3.2	1.1	24.5 ± 3.0	1.1	1.0
Atovaquone	12.7 ± 0.7	12.5 ± 0.5	12.1 ± 0.3	1.0	10.3 ± 0.1	0.8	0.9

Note: etoposide / camptothecin and proguanil were kind gifts from Heidrun Interthal and Akhil Vaidya, respectively; all other compounds were purchased from Sigma.

TABLE S2 Cross resistance characterisation of L262P mutated ATP synthase γ expressing trypanosomes with both endogenous alleles knocked out, with and without (DK) their kinetoplast, compared to WT ATP synthase γ expressing cells.

	Lister 427	γWT	γL262P		γL262P (DK)		
Compound	Mean EC_{50} (nM)	Mean EC_{50} (nM)	Mean EC_{50} (nM)	Resistance Factor *vs.* γWT	Mean EC_{50} (nM)	Resistance Factor *vs.* γWT	Resistance Factor *vs.* γL262P
Miscellaneous trypanocides							
Suramin	69.0 ± 10.9	54.7 ± 11.2	49.0 ± 10.5	0.9	37.9 ± 7.0	0.7	0.8
Nifurtimox	2216 ± 95	2171 ± 36	3931 ± 183	1.8	1974 ± 175	0.9	0.5
Melarsen oxide	9.5 ± 0.4	8.4 ± 0.7	8.4 ± 0.4	1.0	7.5 ± 0.2	0.9	0.9

Note: melarsen oxide was a kind gift from Michael Barrett and Harry de Koning. Suramin was purchased from Sigma.
